# Motif Discovery in Tissue-Specific Regulatory Sequences Using Directed Information

**DOI:** 10.1155/2007/13853

**Published:** 2007-12-24

**Authors:** Arvind Rao, Alfred O Hero, David J States, James Douglas Engel

**Affiliations:** 1 Departments of Electrical Engineering and Computer Science and Bioinformatics, University of Michigan, Ann Arbor, MI 48109, USA; 2 Departments of Bioinformatics and Human Genetics, University of Michigan, Ann Arbor, MI 48109, USA; 3 Department of Cell and Developmental Biology, University of Michigan, Ann Arbor, MI 48109, USA

## Abstract

Motif discovery for the identification of functional regulatory elements underlying gene expression is a challenging problem. Sequence inspection often leads to discovery of novel motifs (including transcription factor sites) with previously uncharacterized function in gene expression. Coupled with the complexity underlying tissue-specific gene expression, there are several motifs that are putatively responsible for expression in a certain cell type. This has important implications in understanding fundamental biological processes such as development and disease progression. In this work, we present an approach to the identification of motifs (not necessarily transcription factor sites) and examine its application to some questions in current bioinformatics research. These motifs are seen to discriminate tissue-specific gene promoter or regulatory regions from those that are not tissue-specific. There are two main contributions of this work. Firstly, we propose the use of directed information for such classification constrained motif discovery, and then use the selected features with a support vector machine (SVM) classifier to find the tissue specificity of any sequence of interest. Such analysis yields several novel interesting motifs that merit further experimental characterization. Furthermore, this approach leads to a principled framework for the prospective examination of any chosen motif to be discriminatory motif for a group of coexpressed/coregulated genes, thereby integrating sequence and expression perspectives. We hypothesize that the discovery of these motifs would enable the large-scale investigation for the tissue-specific regulatory role of any conserved sequence element identified from genome-wide studies.

## 

[1,2,3,4,5,6,7,8,9,10,11,12,13,14,15,16,17,18,19,20,21,22,23,24,25,26,27,28,29,30,31,32,33,34,35,36,37,38,39,40,41,42]
